# Comparison of Accuracy of Arrival-Time-Insensitive and Arrival-Time-Sensitive CTP Algorithms for Prediction of Infarct Tissue Volumes

**DOI:** 10.1038/s41598-020-66041-6

**Published:** 2020-06-09

**Authors:** Lenhard Pennig, Frank Thiele, Lukas Goertz, Kai Roman Laukamp, Michael Perkuhn, Christoph Kabbasch, Marc Schlamann, Gereon Rudolf Fink, Jan Borggrefe

**Affiliations:** 1 Institute for Diagnostic and Interventional Radiology, Faculty of Medicine and University Hospital Cologne, University of Cologne, Cologne, Germany; 2 Philips GmbH Innovative Technologies, Aachen, Germany; 3 Center of Neurosurgery, Faculty of Medicine and University Hospital Cologne, University of Cologne, Cologne, Germany; 4 Department of Radiology, University Hospitals Cleveland Medical Center, Cleveland, OH USA; 5 Department of Radiology, Case Western Reserve University Cleveland, Cleveland, OH USA; 6 Department of Neurology, Faculty of Medicine and University Hospital Cologne, University of Cologne, Cologne, Germany; 7 Cognitive Neuroscience, Institute of Neuroscience and Medicine (INM-3), Research Center Jülich, Jülich, Germany

**Keywords:** Stroke, Stroke

## Abstract

The purpose of this study was to compare the performance of arrival-time-insensitive (ATI) and arrival-time-sensitive (ATS) computed tomography perfusion (CTP) algorithms in Philips IntelliSpace Portal *(v9, ISP)* and to investigate optimal thresholds for ATI regarding the prediction of final infarct volume (FIV). Retrospective, single-center study with 54 patients (mean 67.0 ± 13.1 years, 68.5% male) who received Stroke-CT/CTP-imaging between 2010 and 2018 with occlusion of the middle cerebral artery in the M1-/proximal M2-segment or terminal internal carotid artery. FIV was determined on short-term follow-up imaging in two patient groups: A) not attempted or failed mechanical thrombectomy (MT) and B) successful MT. ATS (default settings) and ATI (full-range of threshold settings regarding FIV prediction) maps were coregistered in 3D with FIV using voxel-wise overlap measurement. Based on an average imaging follow-up of 2.6 ± 2.1 days, the estimation regarding penumbra (group A, ATI: r = 0.63/0.69, ATS: r = 0.64) and infarct core (group B, ATI: r = 0.60/0.68, ATS: r = 0.63) was slightly higher in ATI but the effect was not significant (p > 0.05). Regarding ATI, Tmax (AUC 0.9) was the best estimator of the penumbra (group A), CBF relative to the contralateral hemisphere (AUC 0.80) showed the best estimation of the infarct core (group B). There was a broad range of thresholds of optimal ATI settings in both groups. Prediction of FIV with ATI was slightly better compared to ATS. However, this difference was not significant. Since ATI showed a broad range of optimal thresholds, exact thresholds regarding the ATI algorithm should be evaluated in further prospective, clinical studies.

## Introduction

Computed tomography perfusion (CTP) represents a valuable adjunct to unenhanced CT and CT-angiography (CTA) in the diagnosis of acute ischemic stroke (AIS), playing a vital role in clinical decision making^1–3^. By generating a four-dimensional dataset of the brain, tissue perfusion parameters derived from CTP allow to differentiate between ischemic core and penumbra using selected combinations and thresholds^4^. If CTP shows a prevalent mismatch between ischemic core and penumbra, patients can benefit from mechanical thrombectomy (MT) up to 24 hours after AIS onset^1^. Moreover, an ischemic core volume of 70 ml is regarded as the critical threshold above which patients show a significantly worse clinical outcome; therefore, accurate core and penumbra measurements derived from CTP are essential^5,6^.

For many years, the arrival-time-sensitive (ATS) post-processing algorithm represented the standard for CTP, which was thoroughly tested during the last two decades^4,7–9^. A potential limitation of ATS is its sensitivity to contrast agent arrival delay and dispersion^7,10^, which can be problematic in patients with reduced cardiac output, tandem occlusions, a stenosis upstream of the infarction, or suboptimal contrast enhancement of the scan^11–13^. As a consequence, ATS perfusion results may underestimate cerebral blood flow (CBF) and overestimate mean transit time (MTT), hence resulting in an overestimation of the ischemic core and an underestimation of the penumbra if CBF values in relation to the contralateral hemisphere (CBF_rel) are used^14–16^.

Over the past years, the arrival-time-insensitive (ATI) algorithm has become available for clinical practice and is used in many institutes as diagnostic standard, which aims to address the limitations of ATS. Contrary to ATS, the ATI deconvolution corrects for the arrival delay of the contrast between selected arterial input function and the examined tissue voxel, therefore improving CTP results for above-mentioned patients^17,18^. The majority of available ATI software products provide a time to maximum (Tmax, the time to the maximum of the residue function obtained by deconvolution) instead of a time to peak map (TTP). Tmax represents the contrast agent arrival delay between the arterial input function and the tissue and has proven to surpass delay-corrected parameters such as mean transit time (MTT) and cerebral blood flow (CBF), which determine hypoperfused tissue^19,20^. The recently released version 9 of IntelliSpace Portal *(Philips Healthcare)* enables calculation of CTP maps with ATS and an updated ATI algorithm using this post-processing platform.

There is still an ongoing discussion whether CTP can predict the final infarct volume (FIV) sufficiently and which parameters and thresholds are to be used^4,21,22^.

The objective of this retrospective study was to evaluate the diagnostic performance of the ATI algorithm and to test if ATI provides an equal or even more accurate prediction of FIV than the ATS algorithm in patients with AIS due to large vessel occlusion. Furthermore, we aimed to provide optimal parameters and thresholds for ATI as potential standard settings for the prediction of FIV.

## Materials and Methods

### Patient selection

The local institutional review board approved this retrospective analysis (reference number: 19–1182; Ethikkomission der Medizinischen Fakultät der Universität zu Köln) and waived the need for patient consent. All methods were performed in accordance with the relevant guidelines and regulations.

We reviewed our institutional database consisting of 7122 consecutive patients who received dedicated CT-imaging for AIS (2010–2018). The study included all patients of this consecutive series that met the inclusion and exclusion criteria. Inclusion criteria were: (a) availability of dedicated CT-imaging within 12 hours after symptom onset, (b) complete occlusion of the middle cerebral artery (MCA) in the M1-/M2-segment or terminal ICA-occlusion as shown by digital subtraction angiography (DSA)/CTA, and (c) radiological follow-up by unenhanced CT or magnetic resonance imaging (MRI) within 14 days after initial imaging. Exclusion criteria were: (a) severe motion artifacts of initial/follow-up imaging (n = 9), (b) fully automated warning signals of acquisition quality faults in CTP (n = 6), and (c) any history of previous AIS. Patient records and images were anonymized before image analysis.

Following data were collected from the patients’ medical charts: age, sex, and National Institutes of Health Stroke Scale (NIHSS) score, the latter determined by the neurologist upon admission. The technical success of MT was evaluated using the modified thrombolysis in cerebral infarction score (mTICI), based on postprocedural DSA images^23^. According to whether a MT was performed and the corresponding mTICI score, patients were divided into two groups: A) not attempted/failed (mTICI 0) and B) successful MT (mTICI 2b or 3). In accordance with stroke guidelines, clinically suitable patients received additional intravenous thrombolysis^24^.

### Imaging

Initial stroke imaging was performed using four different CT scanners: Philips iCT (n: 27), Philips Brilliance 6 (n: 21), Philips Brilliance 64 (n: 5) (*Philips Medical Systems*), and Siemens Somatom Definition Flash (n: 1) (*Siemens Healthcare*).

Our standardized institutional imaging protocol consisted of an unenhanced CT, CTA of cervical and intracranial vessels, and CTP of the brain parenchyma. For CTA, an iodine contrast agent (80 ml Accupaque 350 mg/ml; *GE Healthcare*) was injected intravenously with a flow rate of 4 ml/s. Image acquisition was initiated by bolus tracking/triggering on sight in the ascending aorta (delay of 4.5 seconds). Immediately after acquisition of CTA, a second intravenous bolus injection of iodine contrast agent (40 ml Accupaque 350 mg/ml) was administered at a flow rate of 4 ml/s with CTP being performed after a given delay of 4 seconds. No “Jog”- or “Shuttle”-acquisition was applied. On iCT, CTP contained a field of view (FOV) of 8 cm craniocaudally (cc), Somatom Definition Flash showed a FOV of 10 cm cc. Both included a region from the upper frontal lobe to the lower temporal lobe. On Brilliance CTs, the FOV consisted of 4 cm cc, covering the region of the basal ganglia. All scans had the same temporal resolution of 2 seconds with a scan duration of 60 seconds; imaging parameters are given in Table 1.

**Table 1:** CTP protocols of scanners included in the study. cc = craniocaudal. mAs = milliampere-seconds. kVp = kilovoltage peak.

	iCT	Brilliance 6	Brilliance 64	Somatom Definition Flash
Slice thickness, mm	5	6	6	5
Number of slices	16	4	4	20
Total coverage, mm, cc	80	40	40	100
kVp	80	90	80	80
mAs	200	150	150	180

Follow-up imaging was conducted at different CT and MRI scanners: For group A, imaging included diffusion-weighted magnetic resonance imaging (DW-MRI) and unenhanced CT, using a Philips Intera (1.5 Tesla; n: 9), Philips iCT (n: 1), Philips Brilliance 64 (n: 7), Philips Brilliance 6 (n: 4), Siemens Somatom Definition Flash (n: 3), and Siemens Somatom Force (n: 1). Regarding group B, follow-up imaging was obtained solely from DW-MRI, namely with Philips Intera (n: 28) and Philips Panorama 10 (1 T; n: 1).

### Initial imaging post-processing

**Figure 1:**
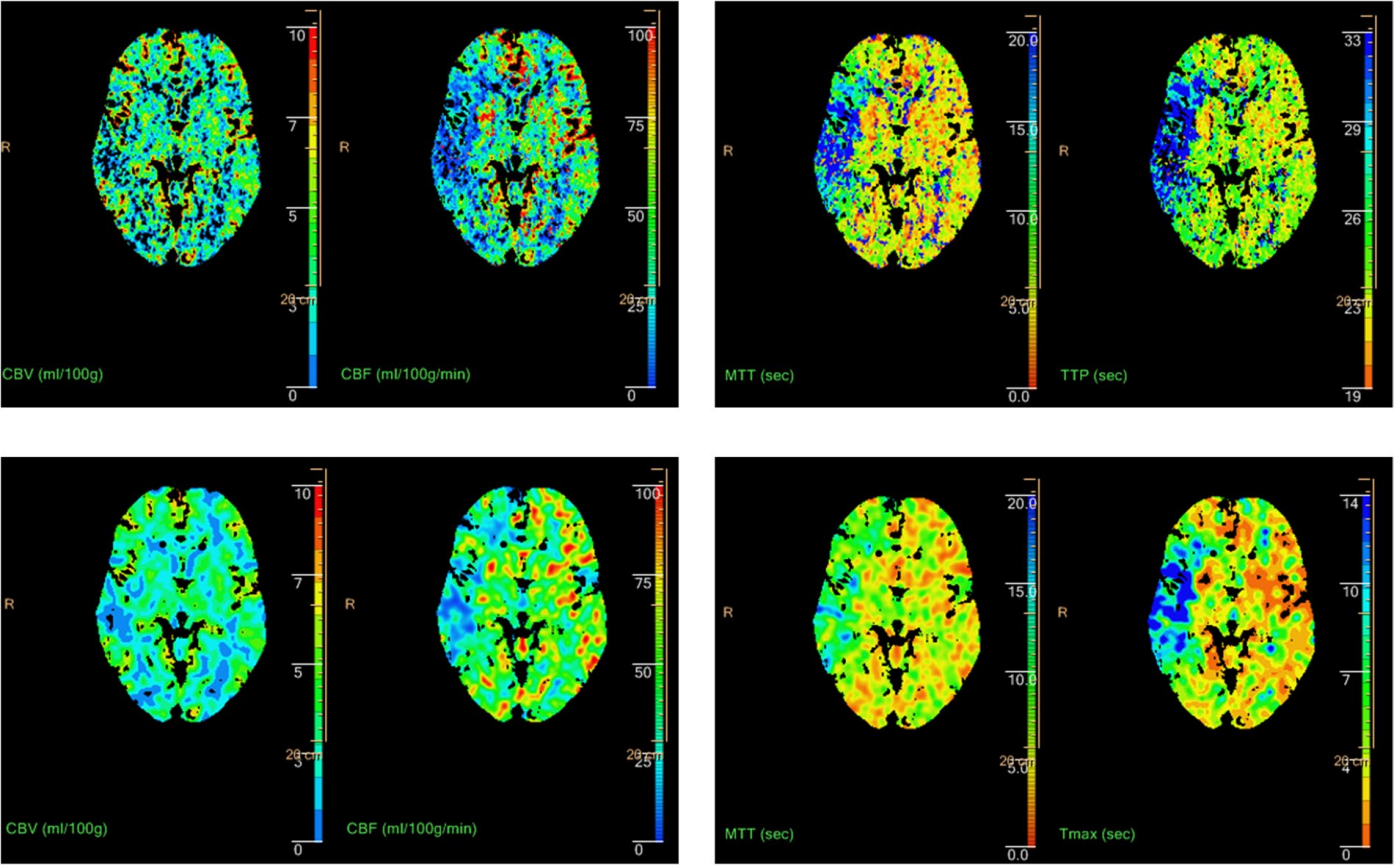
ATS (upper row) - and ATI (lower row) - maps of a patient with a partial but significant CTP-mismatch due to M1-occlusion of the right MCA. CBV = cerebral blood volume. CBF = cerebral blood flow. MTT = mean transit time. TTP = time to peak. Tmax = time to the maximum of the residue function obtained by deconvolution.

CTP post-processing was performed using Communauté Européenne (CE) approved ATS and ATI tools that are both included in the same version of IntelliSpace Portal (ISP,* V9, Philips Healthcare*) (Fig. 1). For both methods, the same automated vessel selection (arteries and veins) was selected as suggested by the perfusion software after verification by the study radiologist and adjusted if necessary.

ATI und ATS parametric maps were computed from CTP data. ATS evaluation used the default threshold settings that are recommended by the vendor (relative MTT >150%, CBV <2 ml/100 g)^4^. In contrast, ATI evaluation was performed for a full range of thresholds.

Resulting parametric maps of ATS and ATI algorithms (Fig. 1) were imported into the Intellispace Discovery (*ISD, v 2.0, Philips Healthcare)* platform for processing. In addition to the four parametric maps, corresponding “relative” parametric maps were computed with voxel values given relative to the corresponding value in the other hemisphere.

### Follow-up imaging and comparison between CTP and FIV

**Figure 2:**
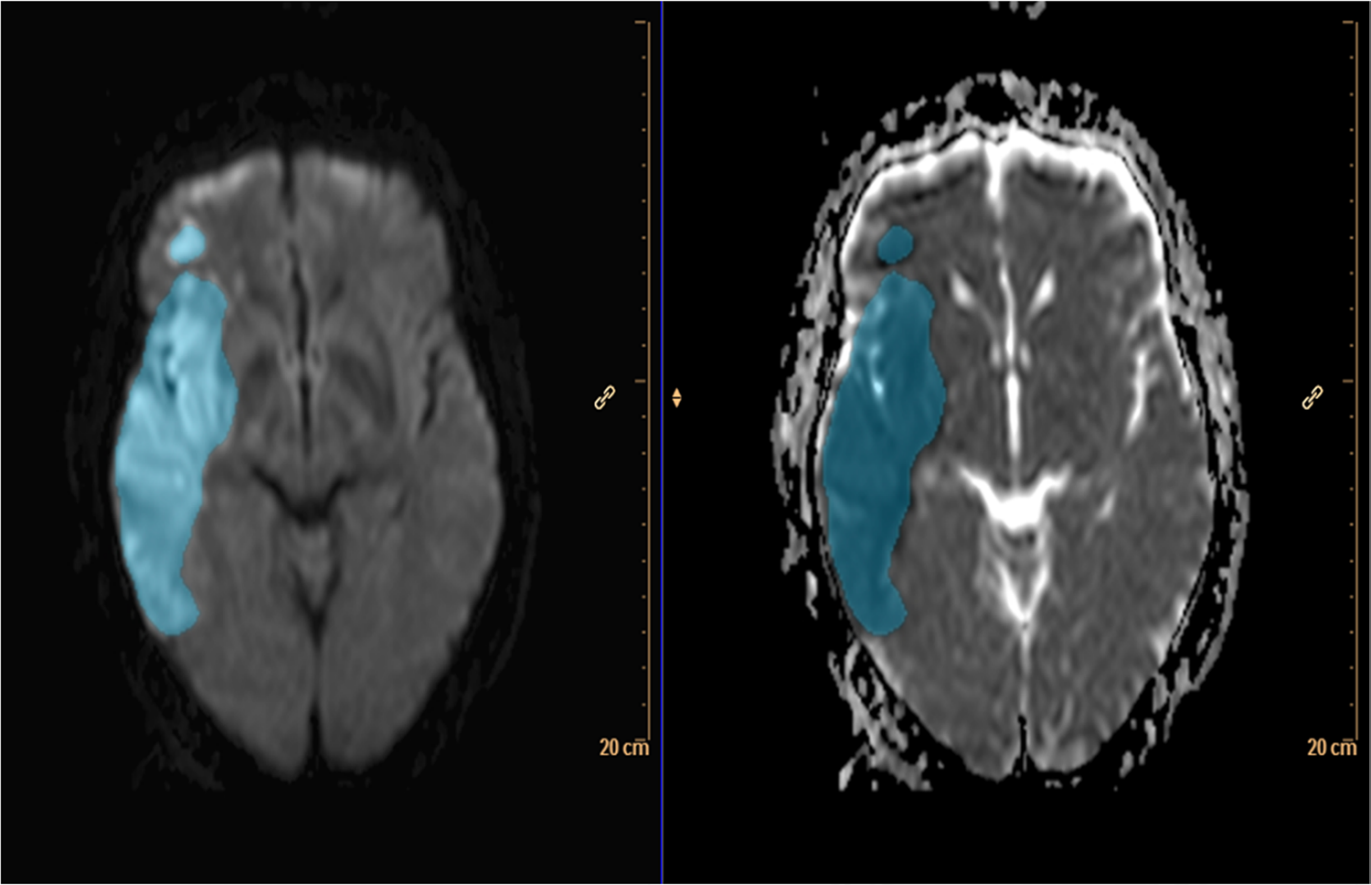
Segmentation of the final infarct volume (FIV) of the same patient as in Fig. 1 based on manual segmentation one day after initial imaging on DW-MRI (left: axial DWI, right: corresponding apparent diffusion coefficient (ADC)-map) after successful MT (mTICI 3, group B).

If both CT and MRI were performed for follow-up imaging, MRI was chosen due to the higher diagnostic sensitivity regarding ischemia in MRI^25^. Imaging data was exported to the ISD platform. In this environment and before creating the CTP maps, the FIVs were manually delineated by a trained and fully blinded radiologist with two years of experience in stroke diagnostics using DWI-images and their corresponding ADC-maps as well as FLAIR (Fig. 2). Thereafter, segmentations were checked and adjusted if necessary by a fully blinded senior consultant neuroradiologist.

In order to allow for a voxel-wise comparison of FIV regions to the CTP parametric maps, for each subject, the follow-up image was automatically co-registered to the CTP study and reformatted to the voxel dimensions of the CTP study. Areas of the brain not covered by CTP thus were not included for comparison of CTP and FIV. The co-registration was based on SPM8 *(Wellcome Trust Centre for Neuroimaging)*. Then, binary maps were created by applying thresholds to the parametric map (standard settings for ATS and full range of settings for ATI). For example, Tmax thresholds were applied from 1 second to 16 seconds with an increment of 0.5 seconds, resulting in 31 binary maps. In each binary map, voxels were set to 1, if Tmax exceeded the threshold at that position, and to zero otherwise. Thresholds used for evaluation of ATI are provided in Table 2.

**Table 2:** Thresholds used for evaluation of ATI with minimum and maximum values as well as applied increment.

Parameter	Minimum	Maximum	Increment
CBV (ml/100 g)	1	10	0.1
MTT (sec)	1	20	0.5
CBF (ml/100 g/min)	1	90	1
Tmax (sec)	1	16	0.5
CBV_rel	0.05	3.5	0.05
MTT_rel	0.05	3	0.05
CBF_rel	0.05	3	0.05
Tmax_rel	0.05	4	0.05

CBV = cerebral blood volume. MTT = mean transit time. CBF = cerebral blood flow. Tmax = time to the maximum of the residue function obtained by deconvolution.

### Statistical analysis

Qualitative discrete variables are presented as numbers and percentages. Quantitative continuous data are shown as mean ± SD, unless noted otherwise. To determine suitable perfusion parameter maps thresholds for ATI, a receiver operating characteristic (ROC) analysis similar to Wintermark *et al*. was applied to the thresholded parametric maps^4^. ROC curves were computed from voxel-wise sensitivity and specificity of overlap with the FIV, each threshold resulting in one point of the ROC curve. To determine an “optimal” threshold, two methods were applied. (I) the Youden Index (sensitivity + specificity -1), and (II) the Dice’s coefficient^26,27^.

The predicted volumes of ATS and ATI were correlated with the FIV using Pearson’s correlation. Wilcoxon tests were performed to compare the following characteristics between both groups: age, male sex, NIHSS core, number of patients who underwent periinterventional thrombolysis, time interval between initial and follow-up imaging as well as the amount of MRI as follow-up and the FIV. Significance was set to p < 0.05. Data analysis was conducted using JMP (*Version 14.1.0, SAS Institute*).

## Results

### Demographics and imaging

54 patients met the inclusion criteria (group A: n = 25, group B: n = 29) (Table 3). The mean patient age of both groups combined was 67.0 ± 13.1 years, being significantly higher in group B (p = 0.0004). The majority of patients was male (68.5%, 37/54) without a significant difference between the groups (p = 0.517). The NIHSS score of all patients combined was 15.3 ± 6.8 without being significantly distinct between both cohorts. Ten patients in group A (40%) and 23 patients in group B (79.3%) received additional intravenous thrombolysis (p = 0.035, 45–90 mg recombinant human-tissue plasminogen activator (rtPA)). In group A, 15 patients (60.0%) received DSA with failed MT (mTICI 0); ten patients did not undergo MT (40.0%).

**Table 3:** Characteristics of both groups with Wilcoxon test being used for comparison of differences, * indicating statistical significance (p < 0.05).

	Overall: (n: 54)	Group A: No/failed MT (n: 25)	Group B: Successful MT (n: 29)	p-value
Age, years, mean ± SD	67.0 ± 13.1	74.0 ± 10.0	61.0 ± 12.0	0.0004*
Male sex, n (%)	37 (68.5)	16 (64.0)	21 (72.4)	0.518
NIHSS, mean ± SD	15.3 ± 6.8	17.1 ± 5.4	14.2 ± 7.3	0.2038
Thrombolysis, n (%)	33 (61.1)	10 (40.0)	23 (79.3)	0.035*
Interval between initial and follow-up imaging, days, mean; SD	2.6 ± 2.7	2.9 ± 2.7	2.4 ± 2.6	0.48
Follow-up imaging with MRI, n (%)	39 (72.0)	10 (40.0)	29 (100.0)	<0.0001*
FIV, ml, mean; SD	48.4 ± 15.16	66.1 ± 68.1	36.5 ± 56.2	0.12

MT = mechanical thrombectomy. SD = standard deviation. NIHSS = National Institutes of Health Stroke Scale. FIV = final infarct volume.

The mean interval between initial stroke CT and follow-up imaging was 2.6 ± 2.7 days (range 0–13 days), being 2.9 ± 2.7 days in group A and 2.4 ± 2.6 days in group B without a statistical difference (p = 0.4825). Fifteen patients (60%) in group A received follow-up imaging on CT; in group B, all patients obtained MRI as follow-up imaging (p < 0.0001). In the whole population, the FIV was 48.4 ± 15.2 ml. The FIV was higher in group A (66.1 ± 68.1 ml) than in group B (36.5 ± 56.2 ml) (p = 0.12).

### Estimation of penumbra (group A)

**Figure 3:**
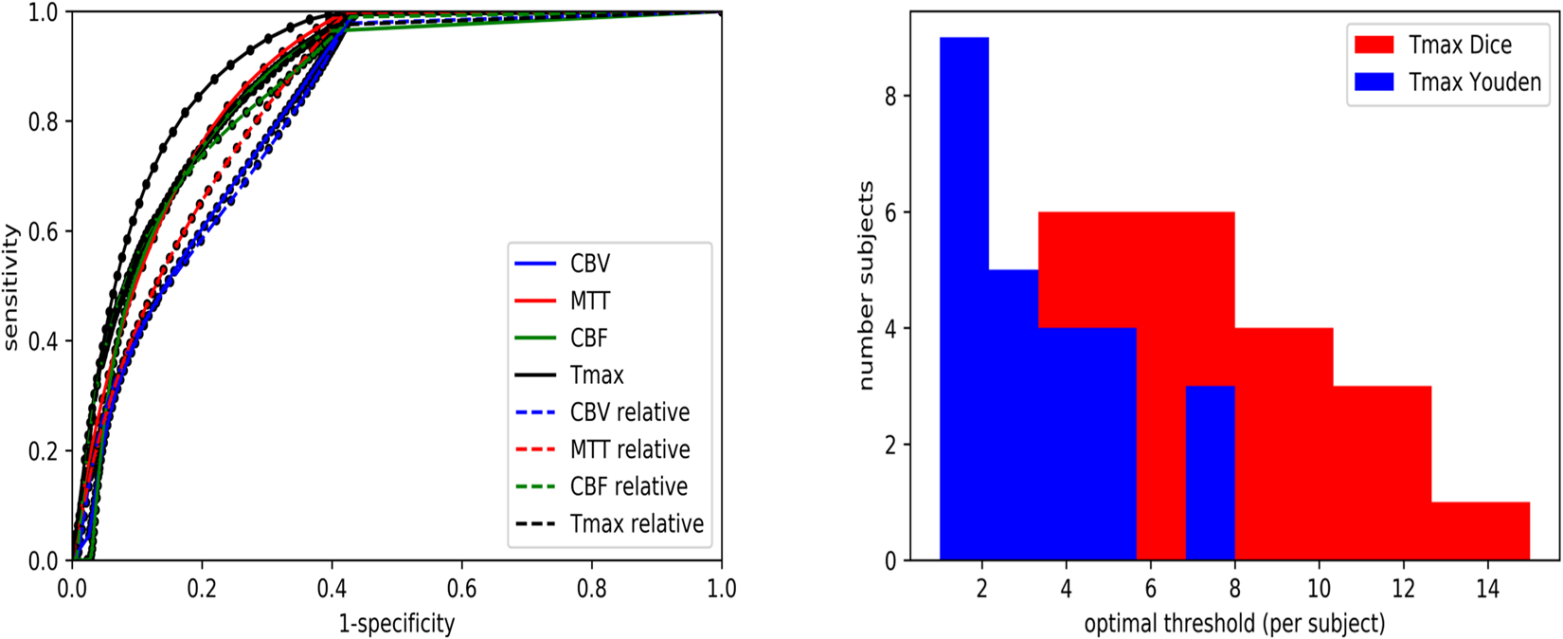
On the left hand side, ROC curves regarding prediction of FIV (group (**A**)) using ATI. On the right hand side, optimal threshold distribution per subject in seconds regarding Tmax of ATI using Dice’s coefficient and Youden-Index. CBV = cerebral blood volume. MTT = mean transit time. CBF = cerebral blood flow. Tmax = time to the maximum of the residue function obtained by deconvolution.

In comparison to ATS (r = 0.63 (factory settings)), ATI provided a slightly higher prediction of FIV with r-values ranging from r = 0.60 (Dice’s coefficient with Tmax >6 seconds; p = 0.3721) to r = 0.68 (Youden-Index Tmax >3.5 seconds; p = 0.1567), albeit this difference was not significant. The area under the curve (AUC) for prediction of FIV in ATI was 0.90 for Tmax, 0.87 for Tmax in relation to the contralateral hemisphere, and 0.87 for MTT, as shown in Fig. 3, when applying a threshold of >6 seconds (using Dice’s coefficient) and >3.5 seconds (using Youden-Index), respectively.

**Figure 4:**
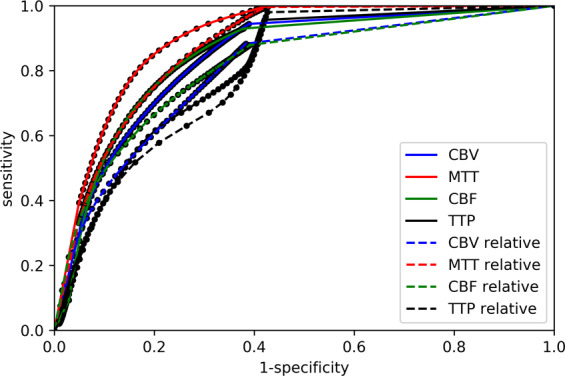
ROC curves regarding prediction of FIV (group A) using ATS. CBV = cerebral blood volume. MTT = mean transit time. CBF = cerebral blood flow. TTP = time to peak.

There was a broad distribution of optimal thresholds for Tmax per subject, ranging from 3–15 seconds (Dice’s coefficient) and from 1–8 seconds (Youden) as illustrated in Fig. 3. Figure 4 depicts ROC curves regarding prediction of FIV in group A using ATS.

### Estimation of infarct core (group B)

**Figure 5:**
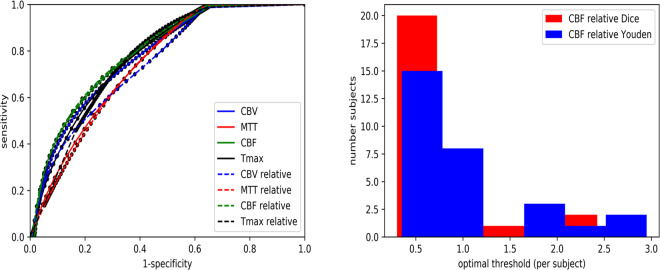
On the left hand side, ROC curves regarding prediction of FIV (group B) using ATI. On the right hand side, optimal threshold distribution per subject regarding CBF_rel of ATI using Dice’s coefficient and Youden-Index. CBV = cerebral blood volume. MTT = mean transit time. CBF = cerebral blood flow. Tmax = time to the maximum of the residue function obtained by deconvolution.

Compared to ATS (r = 0.65 (factory settings)), ATI showed a slightly stronger volume prediction of FIV with r-values ranging from r = 0.63 (Dice’s coefficient with Tmax >6 seconds and CBF in relation to the contralateral hemisphere (CBF_rel <0.75; p = 0.6632) to 0.69 (Youden-Index Tmax >3.5 seconds, CBF_rel <0.6; p = 0.2662). However, there was no statistically significant difference. The AUC for prediction of FIV was 0.80 for CBF_rel, 0.79 for CBF and 0.78 for CBV (by applying a threshold of Tmax >6 seconds), as shown in Fig. 5. When setting a threshold of Tmax >3.5 seconds based on the Youden-Index, Tmax showed the strongest AUC (0.83), and CBF_rel was second best (AUC 0.82).

**Figure 6:**
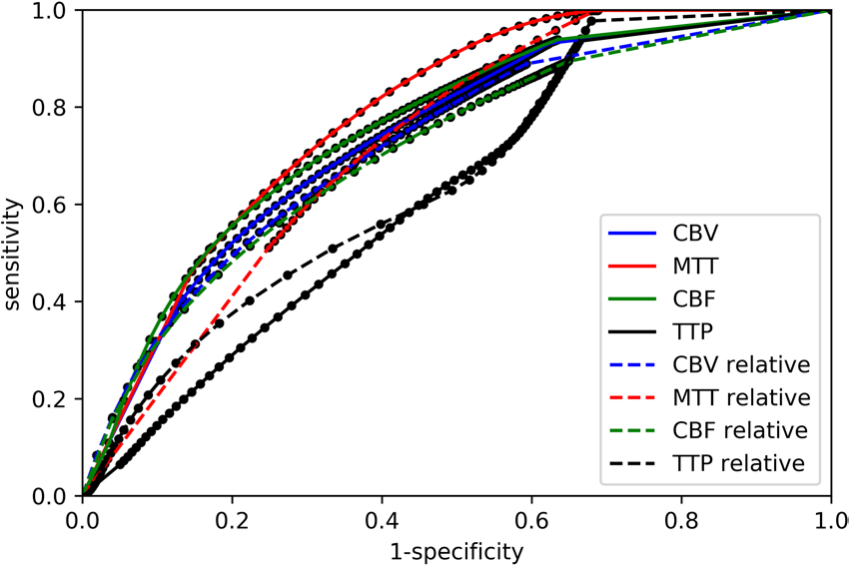
ROC curves regarding prediction of FIV (group B) using ATS. CBV = cerebral blood volume. MTT = mean transit time. CBF = cerebral blood flow. TTP = time to peak.

There was a broad distribution of optimal thresholds for CBF_rel per subject, as demonstrated in Fig. 5. Figure 6 depicts ROC curves regarding prediction of FIV in group B using ATS.

## Discussion

This study tested the performance of ATI for the prediction of FIVs in follow-up imaging and compared the performance to the established reference method ATS. ATI yielded a slightly higher correlation of FIV than ATS without a statistical significance. To our knowledge, this study is the first to compare ATS and ATI algorithms of ISP *V9* for the prediction of FIV in a comprehensive patient cohort with frontal stream occlusion using advanced methods of image coregistration of CTP and FIV.

Investigating the same imaging protocol on four different scanners by different vendors, we provide clinical routine (“real life”) data showing the wide applicability of the two algorithms. Fahmi *et al*. compared an ATS (Philips) and ATI (Siemens) algorithm, but solely in regard to the prediction of penumbra and infarct core without the FIV as reference endpoint^28^. In contrast, our study provides follow-up imaging to correlate perfusion parameters with the true outcome. Man *et al*. conducted a similar study with comparison of ATS and ATI algorithms but used an older version of ISP (*V.4.5.2*)^29^. In contrast, *V9* yields a novel ATI approach; furthermore, no thresholds were evaluated in this previous study.

In comparison to ATS, the ATI CTP algorithm has been shown to reduce dependency on bolus shape and cardiac output, which may have a significant impact on the evaluation of patients with impaired cardiac function or carotid stenosis^30,31^. Our results showed that ATI indeed provided a stronger association with core and penumbra, however not significantly better than ATS. In this regard, our results differ from the findings by Austein *et al*. or Fahmi *et al*., who reported a significant difference regarding prediction of FIV between ATS and ATI^28^,^32^. We assume, that the differences in these prior studies were associated with the use of ATS and ATI algorithms from different vendors^28,32^. Comparable to the study by Man *et al*., we could not detect a statistical significance when comparing both algorithms^29^.

Our results for correlation of initial perfusion imaging and FIVs were comparable to current literature (r-values between 0.58 and 0.78^33^,^34^). With an average value of 66.1 ml, the FIV in patients with none or failed recanalization was lower compared to previous studies. For instance, the FIV was 114 ml in the study by Austein *et al*.^32^ and of 113.4 ml in the study by Flottmann *et al*.^35^. The FIV in patients with successful recanalization was 36.5 ml and thus within the range of the values of the above cited studies, with reported volumes of 38 ml and 35.7 ml, respectively^32,35^. The above-mentioned differences are of importance, since the study by Austein *et al*. showed that each tested CTP algorithm gave false-positive mismatch estimations with FIVs <70 ml, therefore potentially affecting the results in both groups^32^.

In the current study, Tmax is the best performing predictor of penumbra in ATI, thus confirming the results of previous studies^30,32^. The AUC of Tmax for prediction was 0.9, comparable to Campbell *et al*.’s study (AUC 0.87)^36^. In the present study, CBF_rel proved to be the best predictor of infarct core (AUC 0.80), yielding comparable results to Campbell *et al*. (AUC 0.78)^21^ or Yu *et al*. (AUC 0.76)^37^.

Another objective of our study was to determine standard values and thresholds of the ATI algorithm for accurate prediction of FIV for Tmax and CBF_rel. However, there was a broad distribution of optimal thresholds for the estimation of penumbra and infarct core, rendering a definition of one single exact threshold difficult. In contrast, Olivot *et al*. established thresholds for Tmax (>4 and >6 seconds respectively) and Campbell *et al*. for CBF_rel (<31% of the contralateral mean CBF)^21,30^ This difference between our results and previous studies maybe due to the relatively small number of patients in group A (25 in comparison to 33 in Olivots study and 54 in Campbells study or 46 in Wintermarks study regarding ATS) since our study population only consisted of a few cases with frontal stream occlusions that did not receive a successful MT^4,21,30^. Patients in group A were significantly older than in group B, as can be expected since older patients are less likely to be treated by MT^38,39^.

Besides the retrospective setting and the relatively small sample size of group A, our study has several limitations. There were different FOVs of CTP across scanners that need to be considered since the volume of infarct core and penumbra is underestimated when decreasing the coverage^40^. Furthermore, the effects of periinterventional thrombolysis need to be considered since patients in group B received additional thrombolysis significantly more often, potentially affecting the FIV^41–43^. The different follow-up imaging (solely MRI in group B, mixed CT and MRI in group A) and the resulting difference in measuring FIV between both modalities also needs to be considered, especially when measuring the FIV on CT in the first two days after infarction. FIV measurement was conducted up to 13 days after initial imaging. Giving that brain edema after stroke usually peaks at four to five days, it could potentially influence the measurement of FIV, especially on CT, leading to a higher FIV than predicted^44,45^. All in all, infarction pathogenesis is highly variant with each day; therefore we cannot exclude the possibility of infarct progression between different time points of follow-up imaging.

Generally, CTP only offers a temporary view of the confluent processes underlying an ischemic stroke. Volume differences between initial imaging and FIV depend on various factors, such as time to recanalization, collaterals, and edema in follow-up imaging, resulting in a difficult precise prediction of the ischemic core^46^. Furthermore, a recent study showed that thresholded CTP-derived CBF maps could not substitute for a DW-MRI assessment of the size of the ischemic core^47^.

We believe that further larger prospective studies are warranted in order to investigate optimal threshold settings for ATI, especially regarding cases with untreated large vessel occlusion, which are rare since these patients most likely receive a MT. These studies should be established in a clinical setting to determine whether differential results of the algorithms lead to changes in the clinical decision-making. Furthermore, follow-up imaging based solely on MRI should be the method of choice to differentiate between edema and infarct^25^.

In the current study, ATI had a slightly higher predictive value for the final infarct volumes than ATS. However, this difference was statistically not significant. Using ATI, Tmax (penumbra) and CBF_rel (core) were best performing parameters for the prediction of FIV with a large variability of optimal thresholds. Hence, specific thresholds cannot be recommended based on our results. More extensive, prospective studies, maybe in a clinical prospective setting, are warranted to allow in-depth analysis of thresholds for ATI and whether the two algorithms could lead to changes in clinical decision-making.

## Data Availability

The datasets generated during and/or analysed during the current study are not publicly available due to data protection but are available from the corresponding author on reasonable request.
